# Morphometric Characterization of Frontal Sinus and Outflow Tract Anatomy: Implications for Surgical Planning

**DOI:** 10.7759/cureus.112068

**Published:** 2026-07-04

**Authors:** Shrikrishna B H, Deepa G, Mrudula Chandrupatla

**Affiliations:** 1 Otorhinolaryngology Head-Neck Surgery, All India Institute of Medical Sciences, Bibinagar, Hyderabad, IND; 2 Anatomy, All India Institute of Medical Sciences, Bibinagar, Hyderabad, IND

**Keywords:** cadaver, endoscopic sinus surgery, frontal sinus, morphometry, paranasal sinuses

## Abstract

Introduction

The frontal sinus and its outflow tract are integral to sinonasal physiology and play a key role in the development of chronic rhinosinusitis. Their complex anatomy often poses challenges in endoscopic sinus surgery. This study aimed to provide a morphometric characterization of the frontal sinus and its drainage patterns to support safer and more effective surgical planning.

Methodology

A descriptive cross-sectional cadaveric study was conducted on 30 hemisections derived from 15 formalin-fixed cadaveric head and neck specimens at the All India Institute of Medical Sciences (AIIMS) Bibinagar. Frontal sinus height and depth were measured using digital vernier calipers. Drainage routes were traced with a probe in relation to the uncinate process, and anatomical variations were recorded. Data were analyzed using descriptive statistics in Jamovi.

Results

The frontal sinus demonstrated a mean height of 21.3±5.9 mm and a mean depth of 14.9±4.0 mm. Drainage was predominantly anteromedial in 17 specimens (58.6%), followed by anterolateral in eight specimens (27.6%) and posterolateral in four specimens (13.8%). Frontal sinus aplasia was observed in one specimen (3.3%), hypoplasia in two specimens (6.7%), and septation in two specimens (6.7%). One aplastic frontal sinus was excluded from morphometric measurements.

Conclusion

This study shows significant variations in the dimensions and drainage pathways of the frontal sinus. An understanding of frontal sinus anatomy enhances surgical safety and reduces complications.

## Introduction

The frontal sinus and its outflow tract play an important role in the pathophysiology of various sinonasal diseases. Knowledge of this anatomy is important in effective surgical intervention, particularly in endoscopic sinus surgery.

The frontal sinus, an air-filled cavity in the frontal bone, is located behind the brow ridges. It consists of two sinuses on either side that are divided by a bone septum [1]. Frontal, maxillary, ethmoid, and sphenoid sinuses emerge from pouch-like mucosal extensions into the lateral nasal wall [2]. The frontal ostium superiorly and the frontal recess inferiorly are the two parts that make up the frontal sinus drainage channel [3]. This drainage channel is typically shaped like an hourglass. The frontal sinus represents the hourglass's upper part, and its ostium corresponds to its narrowest part. The frontal recess forms the bottom part [4,5]. Endoscopic surgeons are challenged by the complex nature of the frontal sinus outflow tract (FSOT) [6,7]. The present cadaveric anatomical study aimed to analyze the dimensions of the frontal sinus, characterize its drainage patterns in relation to the uncinate process, and document anatomical variations of the frontal sinus and its outflow tract. These findings are intended to provide descriptive anatomical information with potential relevance for endoscopic surgical planning.

## Materials and methods

The study was descriptive and cross-sectional. It analyzed 30 sagittal sections from 15 formalin-fixed cadaveric head and neck specimens. Each cadaveric head was sectioned in the mid-sagittal plane, producing two hemisections. Thus, the study included 30 hemisections derived from 15 cadavers, and observations from left and right sides were treated as paired anatomical specimens. These specimens were obtained from the dissection hall of the Department of Anatomy, AIIMS Bibinagar, India, after approval from the Institutional Research Committee. Since the study exclusively used cadaveric specimens and included no live human or animal participants, it was exempted from Institutional Ethics Committee scrutiny with the IEC Reg. No.: AIIMS/BBN/IEC/JULY/2024-Exemption. Sections with unclear or distorted anatomy that did not show the frontal sinus, outflow tract, or nearby landmarks were excluded from the study. All measurements were performed by a single observer using a calibrated digital vernier caliper. Measurements were repeated twice at separate sittings, and the average value was used for analysis. The digital vernier caliper was calibrated prior to data collection according to the manufacturer's recommendations.

Mid-sagittal sections of the head and neck were considered and the morphometric features, including height and depth of frontal sinus, were carefully observed. Digital vernier calipers were used to record the height and depth dimensions of the frontal sinus. Measurements of the frontal sinus from the highest point to the lowest point indicate the maximum height of the frontal sinus. The distance from the anterior point on the wall of the frontal sinus to the posterior point of the wall of the frontal sinus indicates the maximum depth of the frontal sinus.

To determine the drainage route of the frontal sinus, a probe was inserted into the frontal sinus ostium and traced towards frontal recess. A metallic probe of approximately 3 mm diameter was used to trace the frontal sinus drainage pathway. The probe was carefully moved to explore the drainage pathways and its relationship (anterior, posterior, medial or lateral) to the uncinate process was noted. Once the drainage pattern was established, the entire drainage path of the frontal sinus was exposed and studied. The specimens were also observed for the presence of any anatomical variations related to frontal sinus.

Measurement-related descriptive statistics were performed, such as mean and standard deviation. The frequency and distribution of drainage patterns were analysed categorically.

Statistical analysis was done using MS Excel (Microsoft, Redmond, WA) and Jamovi (open source software, Jamovi Project, Version 2.6.44 for Windows).

## Results

In the present study using 30 cadaveric sagittal sections of head and neck, the following parameters were observed and studied.

Dimensions of the frontal sinus

Morphometric analysis was performed on 29 frontal sinuses with identifiable cavities. One frontal sinus demonstrated aplasia and was excluded from dimensional measurements. Among the 29 measurable sinuses, two sinuses had a height of <10 mm, 10 had a height of 11-20 mm, 15 had a height of 21-30 mm, and two had a height of 31-40 mm. Twenty-five sinuses (86.2%) had a depth of 11-20 mm, while two sinuses (6.9%) each demonstrated depths of <10 mm and 21-30 mm. This distribution highlights that the 11-20 mm depth range was the most prevalent among the studied cadaveric specimens. Specimens demonstrating frontal sinus aplasia were excluded from morphometric measurements because no measurable sinus cavity was present. Of the 30 frontal sinuses examined, one demonstrated aplasia and was therefore excluded from morphometric analysis. The height and depth measurements were calculated for the remaining 29 measurable frontal sinuses.

The mean and standard deviation (SD) for both the height and depth of the frontal sinus were calculated (Table 1). The mean height of the frontal sinus was found to be 21.3 mm with a SD of 5.9 mm, indicating a moderate degree of variability in height among the specimens. The mean depth of the frontal sinus was 14.9 mm with a smaller SD of 4.0 mm, indicating variability comparable to that observed for sinus height. These descriptive statistics provide central tendencies and the spread of the height and depth dimensions of the frontal sinus in the analysed cadaveric specimens.

**Table 1:** Morphometric measurements of frontal sinus. Morphometric measurements were calculated from 29 frontal sinuses. One aplastic frontal sinus was excluded because no measurable cavity was present.

Dimension	Mean (mm)	Standard deviation
Height	21.3	5.9
Depth	14.9	4

Frontal sinus drainage pattern in relation to uncinate process

**Figure 1:**
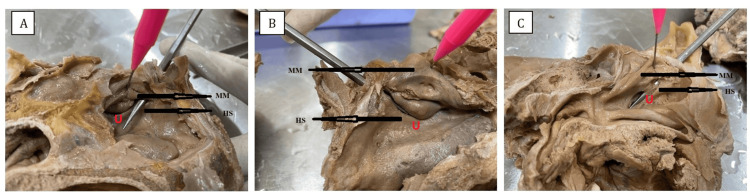
Frontal sinus drainage pattern seen anteromedial (A), anterolateral (B) and posterolateral (C) in relation to uncinate process, as indicated by the probe in a cadaveric dissection. MM: Middle meatus; HS: Hiatus semilunaris; U: Uncinate process.

The frontal sinus drainage pattern was studied in relation to the uncinate process within the studied specimens (Table 2). The most frequent drainage pattern was anteromedial (Figure 1A), found in 17 specimens, representing 58.6% of the sample. Anterolateral drainage (Figure 1B) was the second most common, observed in eight specimens, accounting for 27.6%. Posterolateral drainage (Figure 1C) was less frequent, seen in four specimens, or 13.8% of the total. Notably, in one specimen (3.33%), the frontal sinus drainage was absent or could not be clearly determined in relation to the uncinate process. The specimen showing absent drainage corresponded to the aplastic frontal sinus and was excluded from drainage pattern analysis.

**Table 2:** Frontal sinus drainage pattern in relation to uncinate process. The specimen with frontal sinus aplasia was excluded from drainage pattern analysis because the absence of a sinus cavity precluded identification of a drainage pathway.

S. No	Frontal Sinus Drainage Pattern (In relation to uncinate process)	No. of specimens (n=29)	Percentage (%)
1	Anteromedial	17	58.6
2	Anterolateral	8	27.6
3	Posterolateral	4	13.8

Variations related to the frontal sinus

**Figure 2:**
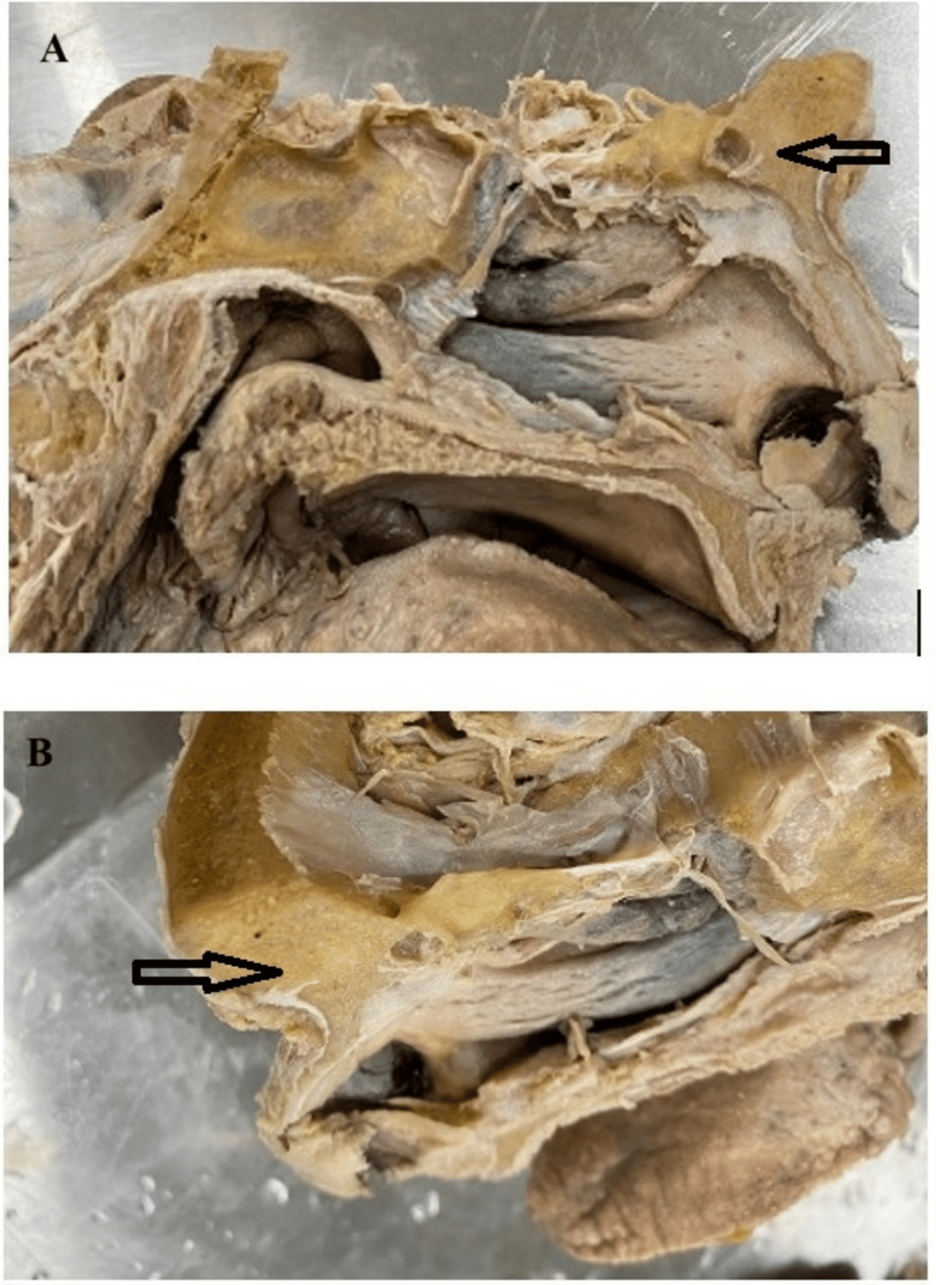
Frontal sinus developmental variations observed in cadaveric dissection include hypoplasia (A), and aplasia (B), both indicated by the black arrow.

**Figure 3:**
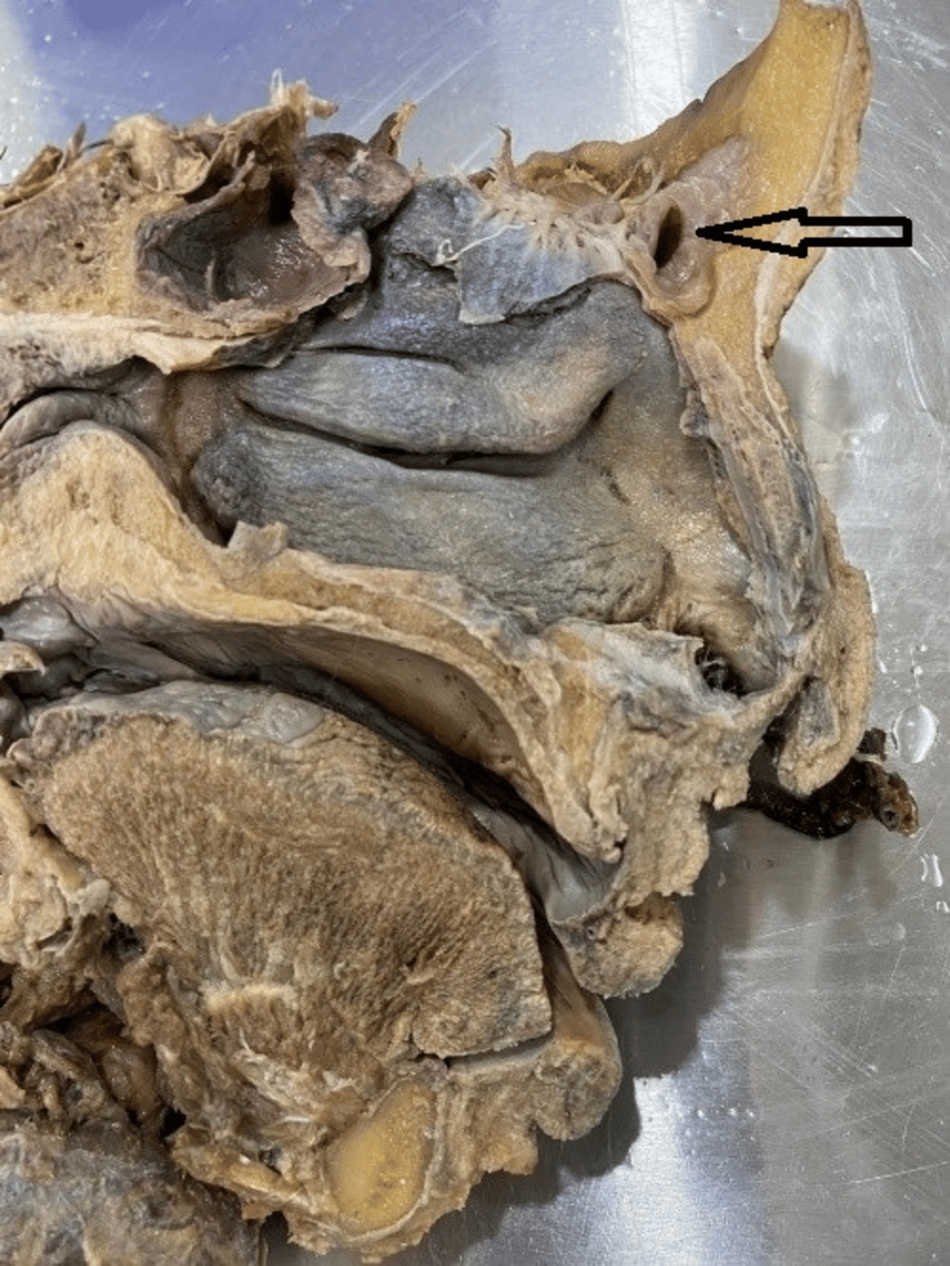
Frontal sinus (FS) with septate cavity (black arrow)

The anatomical variations associated with the frontal sinus cavity were also evaluated (Table 3). Frontal sinus hypoplasia, characterized by incomplete or underdeveloped sinus formation, was identified in two specimens (6.66%) (Figure 2A). Frontal sinus aplasia, defined as the complete absence of the sinus cavity, was observed in one specimen (3.33%) (Figure 2B). The aplastic sinus was observed on the left side. Hypoplasia was observed in one right-sided and one left-sided sinus. In addition, septation within the frontal sinus cavity was noted in two specimens (6.66%) (Figure 3). Overall, these anatomical variations were relatively uncommon within the studied sample, with aplasia, hypoplasia, and septate cavities each occurring in only a small proportion of specimens. The specimen showing absent drainage corresponded to the aplastic frontal sinus and was excluded from drainage pattern analysis.

**Table 3:** Variations of frontal sinus cavity.

S. No.	Type of variation	No. of specimens (n=30)	Percentage (%)
1	Aplasia	1	3.33
2	Hypoplasia	2	6.66
3	Septate cavity	2	6.66

## Discussion

Anatomical variations in the frontal sinus can influence surgical outcomes and require a bespoke approach [8,9]. Our study explored the morphometric characteristics of the frontal sinus, its drainage patterns in relation to the uncinate process (Figure 1), and listed any anatomical variations observed within the frontal sinus (Figure 2).

In our study, the mean height of the frontal sinus was 21.3 mm with a standard deviation (SD) of 5.9 mm, while the mean depth of the frontal sinus was 14.9 mm with a standard deviation of 4.0 mm (Table 1). A CT study by Abdel-Fattah et al. was done on 60 Egyptian adults. It showed a noticeable asymmetry in the frontal sinuses and age-related changes in the width of the right frontal sinus. The study also found a strong positive correlation between the anteroposterior (A-P) lengths of the left and right sinuses [10]. Similarly, research conducted by Gyawali et al. reported that individuals of Nepalese descent had comparatively smaller frontal sinus dimensions than those of other ethnic backgrounds [11].

Our study also explored the pattern of frontal sinus drainage in relation to the uncinate process (Table 2). The anteromedial drainage type (Figure 1A) was the most frequently observed, in 17 specimens, accounting for 56.66% of the specimens. This was followed by anterolateral drainage (Figure 1B) in eight (26.66%) cases, while posterolateral drainage (Figure 1C) was the least common, occurring in only four (13.33%) of specimens. For comparison, Dennis Lee et al. reported that in 29% of specimens, the frontal sinus drained anterior to the uncinate process, whereas 68.3% drained posteriorly [12]. In a related study, Gaafar et al. observed anterior drainage in 23.3%, posterior drainage in 63.3%, and medial drainage into the semilunar hiatus in 6.6% of cases [13]. In the present study, frontal sinus drainage was predominantly anterior to the uncinate process (anteromedial and anterolateral patterns accounting for 86.2% of cases), whereas Lee et al. and Gaafar et al. reported predominantly posterior drainage patterns. This apparent discrepancy may be attributable to differences in the study design, sample size, specimen characteristics, and classification criteria used to define drainage pathways. Furthermore, the present study was based on direct cadaveric dissection, which allows a detailed visualization of anatomical relationships but may differ from observations obtained through radiological or endoscopic assessments. Given the relatively small sample size of the current study, the observed distribution should be interpreted with caution. Nevertheless, these findings highlight the considerable anatomical variability of the frontal sinus outflow tract and underscore the importance of careful preoperative assessment of individual anatomy during surgical planning. 

Additionally, the study examined structural variations of the frontal sinus. Aplasia was noted in one (3.33%) case, while hypoplasia was present in two (6.66%) cases. Septation within the sinus cavity was observed in another two (6.66%) of the samples. Shamlou et al. reported that 3.7% of individuals lacked a frontal sinus entirely, and 12.0% had a unilateral sinus - most commonly on the left side (74.3%) [14]. Our findings are also coherent with findings by Al Hatmi et al., who reported right and left frontal sinus aplasia in 2.1% and 1.8% of cases [15], respectively.

The classification of the frontal sinus anatomy, like the International Frontal Sinus Anatomy Classification (IFAC), provides an ordered scheme for understanding the frontal sinus morphology [16-18]. The use of 3D models and telemedicine systems can also strengthen training in functional endoscopic sinus surgery [18-20]. Studies have also shown the significance of frontal sinus morphology in forensic applications, sex determination, and predicting surgical outcomes [21-23]. This study demonstrates variability in the dimensions and drainage pathways of the frontal sinus. Knowledge of such anatomical variations may assist preoperative assessment and surgical planning, although the present study did not directly evaluate surgical outcomes.

Study limitations

This study is subject to several limitations that should be considered when interpreting the findings. The sample comprised 30 sagittal sections from 15 cadaveric specimens - a modest number that may constrain the broader applicability of our observations. Furthermore, all specimens were sourced from a single institution and had undergone formalin fixation, meaning the anatomical features documented here may not capture the full range of variation present across different populations or preservation conditions. Measurements were obtained from sagittal sections and therefore may not represent the true maximum dimensions of the frontal sinus, which extends laterally beyond the section plane. No formal intra-observer or inter-observer reliability analysis was performed. Measurements obtained from sagittal sections may not represent the true maximum dimensions of the frontal sinus because the sinus extends laterally beyond the plane of section.

The absence of demographic data - including age, sex, and ethnicity - for the specimens studied was an additional constraint, as it prevented any form of subgroup analysis that might have revealed meaningful anatomical differences between groups. Lastly, the study relied solely on direct cadaveric dissection, and no attempt was made to correlate these findings with radiological imaging or postoperative outcomes. Such a correlation, had it been feasible, would likely have strengthened the translational value of the work and offered clearer pathways toward clinical application.

## Conclusions

This cadaveric anatomical study provides descriptive data regarding frontal sinus dimensions, drainage pathways, and anatomical variations that may be useful during preoperative anatomical assessment and surgical planning. As the field of otolaryngology continues to evolve with technological advancements, the significance of understanding the complex anatomy of the frontal recess remains a cornerstone of effective surgical practice.
